# Cardiac Arrest Due to Brugada Syndrome Associated With Influenza Infection: A Case Report and Literature Review

**DOI:** 10.7759/cureus.37158

**Published:** 2023-04-05

**Authors:** Ryohei Ono, Yasuhiko Hori, Tatsuro Yamazaki, Hidehisa Takahashi, Kenichi Fukushima

**Affiliations:** 1 Department of Cardiology, Matsudo City General Hospital, Chiba, JPN

**Keywords:** brugada syndrome, ventricular fibrillation, influenza, fever, cardiac arrest

## Abstract

A 38-year-old Japanese male with no significant medical history but a family history of sudden cardiac death was referred for cardiac arrest. He had a fever (40°C) one day before his visit. His wife reported that he groaned while unconscious, which prompted a referral to the authors’ hospital. He was febrile and experienced ventricular fibrillation in the emergency department. After the resolution of ventricular fibrillation, electrocardiography revealed a right bundle branch block with ST-segment elevation in leads V1-3, consistent with a Brugada electrocardiographic pattern; he also tested positive for influenza A infection. Antiarrhythmic and antipyretic agents were administered, and peramivir was initiated; a fatal arrhythmia did not occur. A cardioverter-defibrillator was implanted, and the patient was discharged without complications. Brugada syndrome is a genetic disease that causes fatal cardiac arrhythmias, with fever recognized to induce the Brugada electrocardiographic pattern. The mechanism of the Brugada-type electrocardiographic pattern, right bundle branch block, and ST-segment elevation in the right precordial leads is considered to be the result of an outward shift of ionic currents during early repolarization, causing a marked abbreviation of the action potential in epicardial cells of the right ventricle. Activation and inactivation kinetics for early sodium currents are faster at higher temperatures. To date, there have only been four published reports describing Brugada-like electrocardiographic changes associated with fever related to influenza infection, and this is the first report of cardiac arrest. Since influenza infection can cause high fever and trigger the fetal arrhythmia of Brugada syndrome, it is important to shorten the duration of the fever. Anti-influenza therapy may be considered in patients who have a history of sudden cardiac arrest in the family, as influenza may influence the development of the Brugada ECG pattern in these individuals. The authors also review the literature on Brugada-like electrocardiographic changes induced by influenza infection. Physicians should be aware that Brugada's electrocardiographic pattern and cardiac arrest can be caused by febrile episodes, including those related to influenza infection.

## Introduction

First described in 1992, Brugada syndrome is a genetic disease associated with a significant risk for fatal cardiac arrhythmias, especially in young men with no evidence of structural heart disease [1]. It is characterized by a right bundle branch block (RBBB), persistent ST-segment elevation, a normal QT interval, and sudden cardiac death [2]. Fever is recognized to induce the Brugada electrocardiographic (ECG) pattern; other known risk factors include sodium channel blockers, tricyclic antidepressants, anesthetics, cocaine, methadone, antihistamines, and electrolyte imbalances [3]. To date, there have been only four cases of Brugada-like ECG changes associated with fever related to influenza infection reported in the English literature, according to the PubMed database [2, 4-6]. To our knowledge, however, the case presented herein is the first of cardiac arrest due to the Brugada syndrome associated with influenza infection. We also reviewed the literature about Brugada-like ECG changes induced by influenza infection.

## Case presentation

**Figure 1:**
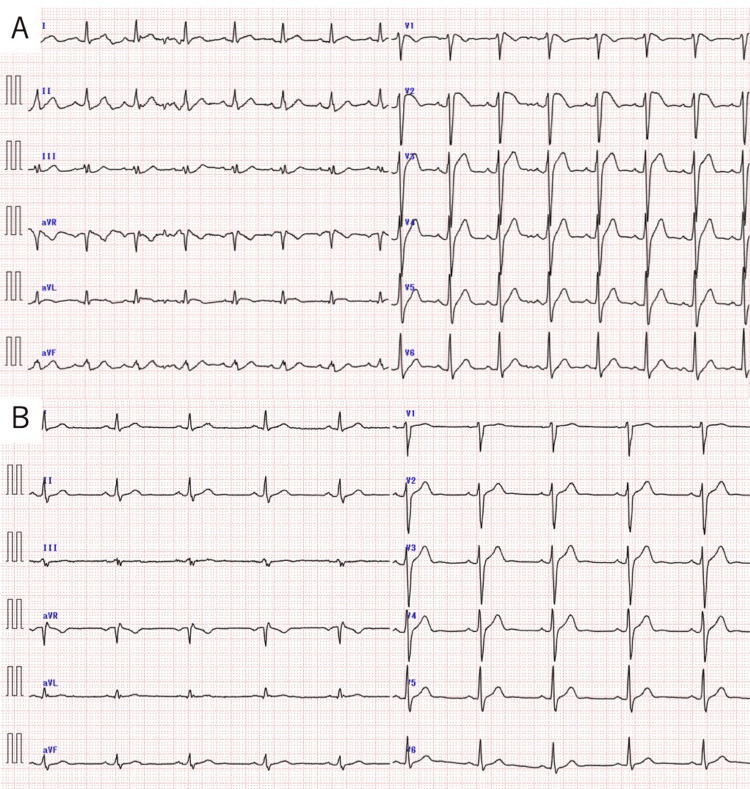
Electrocardiogram (A) The patient’s electrocardiogram, captured during a febrile state of influenza infection, revealed a pattern of right bundle branch block with ST-segment elevation in leads V1-3. (B) Electrocardiogram captured after the resolution of fever confirming the disappearance of coved-type ST elevations.

A 38-year-old Japanese male with no coronary risk factors or cardiac history was referred to our hospital due to a cardiac arrest in January 2020. He had a significant family history of sudden cardiac death: his father and grandfather died at 50 and 57 years of age, respectively. He experienced a fever (40°C) and malaise one day before this visit, for which he took an antipyretic; however, the fever persisted. While the patient was sleeping, his wife recognized that he suddenly groaned without consciousness, which prompted her to call an ambulance. On the arrival of the ambulance crew, he recovered consciousness and was referred to the authors’ hospital. He was drowsy, but his Glasgow Coma Scale score was 15 (E4, V5, M6). He was febrile, with a temperature of 38.8°C, but other vital signs were stable. Physical examination revealed pharyngeal erythema and lymph follicles in the posterior pharyngeal wall but was otherwise unremarkable. However, during a physical examination at the emergency department, he experienced sudden-onset ventricular fibrillation (VF). Fatal arrhythmias had occurred repeatedly in the short-term intermittent period, but regular heartbeats were restored by applying defibrillator pads and shocking the patient with 150 joules on a biphasic defibrillator each time. Electrocardiography revealed RBBB with ST-segment elevation in leads V1-3, consistent with a type-I (coved) Brugada-ECG pattern (Figure 1A). Brugada-ECG also revealed apparent coved-type ST elevations in the right precordial leads. Laboratory investigations revealed elevated white blood cell count and functional liver enzyme levels; however, cardiac enzyme levels, including troponin, creatine kinase (CK), and creatine kinase myocardial band (CK-MB), were within normal ranges. Electrolyte analysis revealed hypomagnesemia (1.7 mg/dL (normal range, 1.8‒2.6 mg/dL)), and the potassium level was 3.9 mEq/L (normal range, 3.5‒5.0 mEq/L). A rapid influenza test was positive for influenza A. Chest radiography yielded unremarkable findings. Cardiac echocardiography revealed a preserved ejection fraction, and no apparent hypertrophy or left ventricular asynergy was noted. Coronary computed tomography was performed to assess possible ischemia and structural abnormalities but revealed no significant stenosis of the coronary arteries and no structural abnormality. The patient was diagnosed with Brugada syndrome induced by fever due to influenza virus A infection. His VF storm was treated with correction for magnesium and potassium, isoprenaline, lidocaine, peramivir for influenza, and acetaminophen. Isoprenaline and lidocaine were administered for four consecutive days. The fever was well controlled by the administration of peramivir and acetaminophen. After the fever subsided, electrocardiography was performed again and confirmed the disappearance of the coved-type ST elevations (Figure 1B).

Genetic testing for Brugada syndrome was not performed because the patient refused to undergo the test. He received an implantable cardioverter-defibrillator (ICD) on day 18 after admission. He was discharged on day 26 without any complications. At the one-year follow-up, he did not experience illness, fever, or any cardiac events and was doing well.

## Discussion

The PubMed database was searched for articles using the terms “Brugada” and “Influenza”. To date, only four case reports have been published in English. The major clinical features described in the four previously reported cases of Brugada-like ECG changes and our case are summarized in Table 1 [2, 4-6].

**Table 1:** Clinical features of Brugada-like electrocardiographic changes associated with fever of influenza infection ND: Not described; ICD: Implantable cardioverter-defibrillator

Case	Author/Reference	Age (years)	Sex	Type of influenza	Body temperature (℃)	Family history	Cardiac arrest	Treatment
1	Kusaka [2]	32	Male	B	39.3	ND	No	None
2	Baranchuk [4]	49	Male	A(H1N1)	38.3	Yes	No	ICD implantation
3	Wang [5]	37	Male	A(H7N9)	39.8	Yes	No	Oseltamivir
4	Kaur [6]	51	Male	B	39.3	No	No	Oseltamivir Acetaminophen
5	Ono, our case	38	Male	A	38.8	Yes	Yes	Peramivir Acetaminophen ICD implantation

The mean age of the patient population was 41.4 years (range, 32‒51 years), and all were male. Influenza was classified as type A in three cases and type B in two; of the three cases, type A, H1N1, and H7N9 were classified as subtypes. The average body temperature on arrival was 39.1°C (range, 38.3‒39.8°C). Three cases had a significant family history of sudden cardiac death. Although all patients exhibited Brugada-like ECG changes, our patient was the first to experience cardiac arrest due to the Brugada syndrome associated with influenza infection. Treatments included anti-influenza agents, such as oseltamivir or peramivir, antipyretic agents, and ICD implantation.

Fever has been reported to induce a Brugada-type ECG pattern [3]. A previous study demonstrated that eight of the 402 patients with fever, but only one of the 909 afebrile patients, had a Brugada-type ECG pattern. Therefore, the prevalence of the Brugada-type ECG pattern in patients with fever was 20 times higher than in afebrile patients, emphasizing the potency of fever in uncovering this ECG phenomenon [7]. The mechanism of the Brugada-type ECG pattern, RBBB, and ST-segment elevation in the right precordial leads is considered to be the result of an outward shift of ionic currents during early repolarization, causing a marked abbreviation of the action potential in epicardial cells of the right ventricle [8]. Activation and inactivation kinetics for early sodium currents are twofold faster at higher temperatures [2]. Mutations in cardiac sodium channel genes, such as SCN5A, have been associated with Brugada syndrome, and dysfunction of the sodium channel is temperature-sensitive in most of the reported mutations, thus suggesting the possibility that the ECG features of the syndrome may be enhanced during febrile states [9]. Although influenza infection can cause high fever, a relationship between Brugada syndrome and influenza infection itself has not been established in previous studies. However, since influenza infection can cause a high fever and trigger the fetal arrhythmia of Brugada syndrome, it is important to shorten the duration of the fever. A previous study reported the remission time of fever symptoms in the peramivir group was 12 hours, which was significantly shorter than that in the oseltamivir group (24 hours); therefore, our case used peramivir [10]. Anti-influenza therapy may be considered in patients who have a history of sudden cardiac arrest in the family, as influenza may influence the development of the Brugada ECG pattern in these individuals. Our review also suggests that a temperature > 38°C may be a risk factor for the Brugada ECG pattern based on the cases described above.

The treatment of Brugada syndrome is mainly focused on the prevention of sudden cardiac arrest. ICD implantation is the first-line therapy for the termination of ventricular arrhythmias. ICD is indicated for those who have survived sudden cardiac arrest, as in this case, or those with a history of the syndrome from ventricular arrhythmias. However, treatment for asymptomatic patients with a Brugada ECG pattern but no history of syncope, arrhythmia, or family history of cardiac death has not been established. Electrophysiological studies in these patients may be beneficial to evaluate risk [4, 6].

## Conclusions

In conclusion, we report a rare case of cardiac arrest in a young male with Brugada syndrome associated with a fever related to an influenza infection. Physicians should be aware that the Brugada ECG pattern and cardiac arrest can be caused by febrile episodes, including those related to influenza infection.
